# IUC26679-95 A pilot study of the implementation of the G8 frailty assessment in oncology clinics for patients with newly diagnosed metastatic hormone-sensitive prostate cancer

**DOI:** 10.1093/oncolo/oyag286.015

**Published:** 2026-08-21

**Authors:** A Hague, S Appleyard, A Balagopal, N Samkaoui

**Affiliations:** East Sussex Healthcare Trust, East Sussex - United Kingdom; University Hospitals Sussex, Brighton and Hove - United Kingdom; University Hospitals Sussex, Brighton and Hove - United Kingdom; University Hospitals Sussex, Brighton and Hove - United Kingdom

## Abstract

**Background:**

Treatment intensification for patients with metastatic hormone-sensitive prostate cancer (mHSPC) is now recommended as standard of care with either doublet or triplet therapy. The 2025 National Prostate Cancer Audit showed 47% men with mHSPC received treatment intensification in England. We audited our own practice over 1 year and found that 70% of patients with mHSPC were offered treatment intensification. A 2024 systematic review found that younger patients with fewer comorbidities were more likely to receive treatment intensification. More comprehensive frailty assessments are being adopted to appropriately escalate treatment within oncology. The Geriatric 8 (G8) screening tool allows for a more efficient assessment and referral for a comprehensive geriatric assessment. Primary endpoint: To assess the feasibility of implementation of the G8 screening tool within a uro-oncology clinic in a District General Hospital (DGH) as part of standard practice. Secondary endpoint: To ascertain whether there is a threshold for the G8 score which corresponds with those patients who were offered additional upfront treatment within the pilot study.

**Methods:**

A 6-month pilot study was carried out at our local DGH. Patients with newly diagnosed mHSPC referred to oncology were included. Patients were identified through the MDT. The G8 screening tool was completed at the first oncology appointment by a healthcare professional (HCP). The HCP who completed the G8 tool was then asked to complete a questionnaire to assess the acceptability of the intervention.

**Results:**

Over 6 months, 28 patients met the eligibility criteria for the pilot study. 79% were offered treatment intensification (n  =  22). 68% of the patients offered treatment intensification were under the age of 75 (n  =  15). The G8 scores ranged from 5 – 17 (median = 15). There was a statistically significant association between the G8 screening score and whether patients were offered treatment intensification (Fisher’s exact test p  =  0.00055). The calculated general acceptability mean score was 4 out of 5.

**Conclusions:**

Patients with a high G8 score (14-17) were more likely to be offered treatment intensification compared to those with a low G8 score (<10). HCPs who completed this questionnaire deemed implementing the G8 screening tool to be acceptable.

